# Aquaporin 4 is not present in normal porcine and human lamina cribrosa

**DOI:** 10.1371/journal.pone.0268541

**Published:** 2022-06-16

**Authors:** Elizabeth C. Kimball, Sarah Quillen, Mary E. Pease, Casey Keuthan, Aru Nagalingam, Donald J. Zack, Thomas V. Johnson, Harry A. Quigley

**Affiliations:** Glaucoma Center of Excellence, Wilmer Eye Institute, Johns Hopkins University, Baltimore, Maryland, United States of America; Transilvania University of Brasov: Universitatea Transilvania din Brasov, ROMANIA

## Abstract

Aquaporin 4 is absent from astrocytes in the rodent optic nerve head, despite high expression in the retina and myelinated optic nerve. The purpose of this study was to quantify regional aquaporin channel expression in astrocytes of the porcine and human mouse optic nerve (ON). Ocular tissue sections were immunolabeled for aquaporins 1(AQP1), 4(AQP4), and 9(AQP9), myelin basic protein (MBP), glial fibrillary acidic protein (GFAP) and alpha-dystroglycan (αDG) for their presence in retina, lamina, myelin transition zone (MTZ, region just posterior to lamina) and myelinated ON (MON). Semi- quantification of AQP4 labeling & real-time quantitative PCR (qPCR) data were analyzed in retina and ON tissue. Porcine and control human eyes had abundant AQP4 in Müller cells, retinal astrocytes, and myelinated ON (MON), but minimal expression in the lamina cribrosa. AQP1 and AQP9 were present in retina, but not in the lamina. Immunolabeling of GFAP and αDG was similar in lamina, myelin transition zone (MTZ) and MON regions. Semi-quantitative AQP4 labeling was at background level in lamina, increasing in the MTZ, and highest in the MON (lamina vs MTZ, MON; p≤0.05, p≤0.01, respectively). Expression of *AQP4* mRNA was minimal in lamina and substantial in MTZ and MON, while *GFAP* mRNA expression was uniform among the lamina, MTZ, and MON regions. Western blot assay showed AQP4 protein expression in the MON samples, but none was detected in the lamina tissue. The minimal presence of AQP4 in the lamina is a specific regional phenotype of astrocytes in the mammalian optic nerve head.

## Introduction

A major site of axonal injury to retinal ganglion cell axons in experimental monkey [1] and human glaucoma [2] is the optic nerve head (ONH) at the level of the sclera, the lamina cribrosa. Similar signs of axonal injury and axonal transport blockage are seen in the corresponding unmyelinated optic nerve region in mouse and rat eyes [3]. Astrocytes line the connective tissue beams of the lamina cribrosa in large mammal and human eyes [4]. In rodents, astrocytes span the analogous region, but without collagenous connective tissue support [5]. Both rodent and primate ONH astrocytes are connected to the peripapillary sclera via their basement membrane (BM), reinforced by intracellular junctional complexes just inside the cell membrane. The responses of ONH astrocytes to pathologic insult are likely to be important in glaucoma pathogenesis [6, 7].

In the ONH and brain, astrocytes provide nutrient support to axons from local capillaries. This neurovascular unit allows molecules to reach axons by passing sequentially through capillary cells, capillary BM, perivascular collagenous tissue, astrocyte BM, and astrocytic processes that segregate axons from the vascular spaces. Both nutrient delivery and extracellular fluid content and flow are managed by brain astrocytes using transmembrane channels, including monocarboxylate transporters [8], glucose transporters [9], and aquaporin (AQP) channels [10]. Indirect clinical and epidemiological evidence implicates nutritional deficit as important in glaucoma pathogenesis [11]. but the linkage between mechanical stress and alteration in ONH nutrient provision and fluid equilibrium has not been conclusively determined.

AQP channels, which are transmembrane tetramers of aquaporin subunits, that form full channels only in the presence of α- and β-dystroglycan (DG) membrane complexes [12, 13]. DGs are linked to extracellular glycoproteins, such as agrin, and connect intracellularly to the cytoskeleton through α1-syntrophin [14, 15]. The aquaporins expressed in astrocytes include AQP1, AQP4, and AQP9 (aquaglyceroporin) [16–18]. AQP1 and 4 channels are selectively permeable to water and dissolved gases, while AQP9 is also permeable to glycerol and small uncharged solutes. In brain, astrocyte membranes facing the pia mater, axons, and capillary connective tissue have AQP channels [19–21]. Brain astrocytes express AQP4 in both normal and pathological states, while AQP1 and 9 are expressed in pathological conditions [22, 23].

Interestingly, AQPs are not present in the unmyelinated portion of the ONH in normal mouse [24, 25] and rat [18], nor in the lamina cribrosa of the dog [26], though they are found in retinal astrocytes of these species, as well as in the prelaminar ONH and the myelinated region of the optic nerve. Genetic deletion of AQP4 leads to increased brain extracellular volume [27] and AQP4 was reported to be downregulated in the optic nerve in rat glaucoma [28]. Unfortunately, many prior AQP investigations refer only to studies of the “optic nerve”, but fail to indicate which region of the nerve was studied. Many previous studies included adjoining tissues of prelaminar retina and myelinated nerve in studies under the presumption that expression of most molecules by astrocytes would be similar across these local regions [29, 30]. We have recently demonstrated that there is a highly regional phenotype of astrocytes of the unmyelinated optic nerve zone of mouse that differs from that of their prelaminar nerve head and the myelinated optic nerve [31–33].

Hypothetically, glaucoma [34] or aging [35] in the optic nerve may involve abnormal AQP function that damages axons through altered ONH volume or fluid flow. Recently, fluid movement via a putative glymphatic pathway [36] through the optic nerve was described. It was suggested that cerebrospinal fluid entry into the optic nerve might be impaired in glaucoma [37]—presumably due to alterations in AQP channel function. While Immunolabeling of human secondary glaucoma eyes found reduced AQP9 in the retina, the presence or change in AQP4 in the ONH itself was not demonstrated [38]. We exposed C57BL/6 and AQP4 knock out mice to bead-induced intraocular pressure (IOP) elevation for 3 days, 1 and 6 weeks [39]. Wild type mice had abundant AQP4 expression in Müller cells, astrocytes of the retina and myelinated optic nerve, but minimal AQP4 in prelaminar and unmyelinated optic nerve by immunolabeling and gene expression, despite the presence of the DG complex. Myelinated optic nerves of AQP4 nulls had a lower proportion of nerve tissue occupied by astrocyte cytoplasm. In control mice, AQP4 remained absent in the optic nerve head and unmyelinated nerve after IOP elevation for 3 days. At baseline AQP1 and AQP9 were present in retina, but neither in the unmyelinated nor myelinated nerve, and their distribution was unaffected by IOP elevation. After IOP elevation for up to 6 weeks, axon loss occurred equally in control and AQP4 null mice, indicating that lack of AQP4 was neither protective nor detrimental to the effects of IOP elevation.

We hypothesize that the normal lack of AQP4 at the site of typical glaucoma damage in all animals yet studied is likely to be an evolutionarily conserved, regionally specific phenotype of ONH astrocytes. In the present studies, to extend our prior work, we evaluated expression of AQP1, 4, and 9 in porcine and human glia of the retina, ONH, and myelinated optic nerve using semi-quantitative immunolabeling and real-time quantitative PCR (qPCR).

## Materials and methods

### Tissue

We studied 3 human eyes (age range: 65–78 years old) donated to eye banks and National Disease Research Interchange with no history of eye disease and 18 young (6–9 months old) porcine eyes purchased from a slaughterhouse (Animal Technologies Inc., Tyler, TX). The human eyes were confirmed as having no significant axon loss, nor other sign of disease. The use of post-mortem human tissues qualifies as exempt from Institutional Review Board approval or consent (Revised Common Rule, Exemption 4). Appropriate USDA regulations for animal sacrifice were followed by Animal Technologies Inc., and post-mortem specimens were exempt from Animal Care and Use Committee review.

### Tissue preservation

Porcine globes were received on ice within 18 hours after enucleation (20–24 hours *post mortem*). Muscle, fat and other tissue were removed from the globe to expose the ONH and optic nerve. A small incision was made in the posterior segment at the equator. Porcine eyes were fixed by immersion for 3 hours in 4% paraformaldehyde. Human eye tissues were received on ice within 24 hours after enucleation. Samples were either shipped in phosphate buffer solution (PBS), or in a variety of aldehyde-based fixatives prior to receipt in our laboratory. If samples were shipped in aldehyde-based fixatives, the tissue was then placed in Sorensen’s phosphate buffer (0.1M PO_4_ buffer) upon arrival. PBS-shipped material was fixed on arrival in 4% paraformaldehyde for 12 hours.

Porcine and human eyes were either prepared for epoxy-embedding or cryopreservation. After initial fixation, the anterior segment including the lens was removed. Transverse sections from human ONs were cut 1–3 mm behind the globe and saved for axonal assessment. The posterior globe with initial optic nerve segment was saved for preservation.

Tissue that was processed for cryopreservation was placed in ascending concentrations of sucrose in 0.1M PO_4_ buffer followed by embedding in 2 parts 20% sucrose buffer to 1 part Optimal Cutting Temperature compound (OCT; Sakura Finetek USA. Inc., Torrance, CA). Samples were frozen with dry ice cooled 2-methylbutane and stored at -80 ° C until sectioning in either longitudinal or cross-section orientation.

### Immunolabeling

Posterior poles were cryosectioned in longitudinal or transverse orientation 10 μm thick for human samples, and 12 μm for porcine, then collected onto Superfrost Plus slides (Fisher Scientific; Pittsburgh, PA) for storage at –80°C before immunolabeling. Antibodies used here are given in Table 1. Sections were blocked with normal goat serum (NGS, 2–10%)/ 0.1% BSA or normal donkey serum (NDS, 2%)/ 0.1% BSA in PBS for 30 minutes, rinsed and co-incubated with primary antibody overnight at 4°C. Tissues were washed and secondary antibody applied at 1:500 with 4’-6-diamidino-2-phenylindole (DAPI: Cat # 10-236-276-001, Roche Diagnostics, Indianapolis, IN) at 1:1,000 for 1 hour, followed by washing and mounting in DAKO mounting media (cat# 23023, Agilent Technologies, Santa Clara CA).

**Table 1:** Primary and secondary antibodies.

Primary Antibody	Company	Catalog	Species	Dilution	Mono or Polyclonal
α-Dystroglycan	Sigma	05–593	Mouse	1:200	Monoclonal
Aquaporin 1	Abcam	ab15080	Rabbit	1:500	Polyclonal
Aquaporin 4 [39]	Alomone Labs	249–323	Rabbit	1:500	Polyclonal
Aquaporin 9	Millipore	AB3091	Chicken	1:500	Polyclonal
DAPI	Roche	D9542	Stain	1:10,000	
Glial Fibrillary Acidic Protein	Invitrogen	13–0300	Rat	1:1000	Monoclonal
Integrin beta 1	Abcam	AB183666	Rabbit	1:250	Polyclonal
Myelin Basic Protein	Abcam	ab218011	Rabbit	1:200	Monoclonal
β-actin	Santa Cruze	SC-4777	Mouse	1:5000	Monoclonal
**Secondary Antibody**	**Company**	**Catalog #**	**Species**	**Dilution**	
Alexa-Fluor 488	Invitrogen	A11008	Goat anti-Rabbit	1:500	
Alexa-Fluor 488	Invitrogen	A11008	Goat anti-Rat	1:500	
Alexa-Fluor 488	Invitrogen	A32816	Donkey anti- Rabbit	1:500	
Alexa-Fluor 555	Invitrogen	A21206	Donkey anti-Goat	1:500	
Alexa-Fluor 568	Invitrogen	A11004	Goat anti-Mouse	1:500	
Alexa-Fluor 647	Invitrogen	A32933	Goat anti-Chicken	1:500	
Alexa-Fluor 647	Invitrogen	A21244	Goat anti-Rabbit	1:500	

### Confocal microscopy

Cryopreserved images were produced on the Zeiss LSM 710 microscope (Carl Zeiss Microscopy, LLC, Thornwood NY) using the Plan-Apochromat 20x/0.8 M27 objective, 40x Plan Apochromat, or the 63x 1.40 NA Plan Apochromat with individual tracks for each laser line (405, 488, 555, 568 and/or 647 nm). Images of the ONH in longitudinal or cross section were collected as tiles or as individual frames using the optimal resolution setting of 1940 x 1940 per frame. Tiles were stitched using Zeiss Zen software.

### Semi—Quantification of aquaporin 4 fluorescence

We used our published method for semi-quantifying fluorescence [39] using the pixel intensity value (PIV), calculated using FIJI (ImageJ, Bethesda MD). Longitudinal, cryopreserved porcine sections from 4 samples immunolabeled against AQP4 were imaged using the same acquisition settings. Six regions of interest (ROI) were: choroid, sclera, retina (two regional measurements were taken then averaged), lamina cribrosa, myelin transition zone (MTZ, the ~100 μm long region in which myelination of axons is partially present), and myelinated optic nerve (MON). Mean, median, maximum and minimum values were acquired, and mean values from each sample at each ROI were used to calculate the average ROI for each location.

### Gene expression

Porcine globes processed for qPCR were shipped in PBS on ice, within 18 hours after enucleation (20–24 hrs *post mortem*). Muscle, fat and other tissue were removed from globe to expose the ONH and optic nerve. All tissues were kept on ice during the remaining dissections. Using a razor blade, an incision was made at the equator to remove the anterior segment including the lens. The retina was gently removed using a flat spatula, with a cut made at the ONH to detach the retina. The optic nerve was then dissected from the retina, through the pre-lamina, to the lamina and myelinated nerve. The dura mater was removed along with the peripapillary sclera attached to the ONH. Using a razor blade, the remaining retina and pre-lamina area were separated from the lamina anteriorly. Then, a 0.2mm long cylinder containing the lamina was separated from the myelin transition zone and myelinated nerve with a razor cut. The lamina in the pig has a grossly visible porous connective tissue structure, and in addition there are pigmented cells in its connective tissue beams. Myelin is easily visible as white matter. A second cylinder, 0.1mm in length, was removed from the cylinder behind the lamina and discarded to avoid mixing lamina tissue with areas more posterior to it. A 1.5mm cylindrical sample was then removed and labeled the myelin transition zone, followed by a 3.0mm long sample labeled as myelinated optic nerve. Tissues were placed in Eppendorf tubes that were flash frozen in liquid nitrogen for 10 minutes prior to storing at -80°C until gene expression studies.

Each tissue sample was homogenized in TRIzol Reagent (Thermo Fisher) for RNA isolation according to the manufacturer’s protocol. Volumes were appropriately adjusted for each tissue type (~1mL TRIzol per 50-100mg tissue sample). Purified RNA was synthesized into cDNA using a High Capacity cDNA Reverse Transcription kit (Applied Biosystems) using the manufacturer’s protocol. qPCR was set up in triplicate using SsoAdvanced Universal SYBR Green Supermix (Bio-Rad) and efficiency-validated primer sets that span exon boundaries (Table 2). All primer sets used in this study were validated for performance prior to use in experiments. Amplification efficiencies for each primer set were obtained through generation of a standard curve from a serially-diluted porcine cDNA template. Primer efficiency scores (presented as a percentage) were calculated using the slope value of the regression between the log values and average Ct values of the dilution series (Table 2). A melt curve analysis was also performed following amplification to confirm presence of a single amplicon.

**Table 2:** Primer sequences and amplification efficiencies for qPCR.

Gene	Forward Primer (5’-3’)	Reverse Primer (5’-3’)	Source	Efficiency
*PPIA*	AGGTTCCTGCTTTCACAGAATA	CATAGATGGACTTGCCACCA	This paper	89.1%
*HPRT1*	CCAGTCAACGGGCGATATAA	GACCAAGGAAAGCAAGGTTTG	This paper	80.9%
*HBMS*	AGGAGTTCAGTGCCATCATC	CTGACCCACAGCATACATACA	This paper	110.2%
*CD68*	AGAGCACTGTCTACCTGAACTA	TGGAGATCTCGAAGGGATGAA	This paper	112.9%
*AQP4*	CCCGCAGTTATCATGGGAAA	CCACATCAGGACAGAAGACATAC	PMID: 33529207	97.1%
*GFAP*	CAGAGGAGTGGTATCGGTCTAA	GATAGTCGTTAGCTTCGTGCTT	PMID: 33529207	99.2%

*PPIA-* Peptidylprolyl Isomerase A (housekeeping), *HPRT1*- hypoxanthine phosphoribosyltransferase 1 (housekeeping), *HBMS-* Hemoglobin Subunit Mu (housekeeping), *CD68- CD68* (macrophage/microglia marker), *AQP4-* aquaporin 4 (astrocyte membrane), *GFAP-* glial fibrillary acidic protein (intermediate filaments of astrocytes).

qPCR was performed on a CFX384 Touch Real-Time PCR Detection system with the following conditions: 95°C for 30s, followed by 40 cycles of 95°C for 5s and 60°C for 30s. Relative expression was calculated using the 2-ΔCt method where each raw value for a sample was normalized to the geometric mean values of three housekeeping genes (PPIA, HPRT1, HBMS). An ordinary One-way ANOVA with Tukey’s test for multiple comparisons was performed to obtain p-values on the averaged, normalized data, where a p-value < 0.05 was considered statistically significant.

### Western blot analysis

Nine porcine globes processed for western blot were shipped in PBS on ice, within 18 hours after enucleation (20–24 hrs post mortem). Muscle, fat and other tissue were removed from globe to expose the ONH and optic nerve. All tissues were kept on ice during the remaining dissections. Using a razor blade, an incision was made at the equator to remove the anterior segment including the lens. The retina was removed using a flat spatula, with a cut made at the ONH to detach the retina. The dura mater was removed along with the peripapillary sclera attached to the ONH. Using a razor blade, the remaining retina and pre-lamina area were separated from the anterior lamina, and discarded. Then, a 0.2mm long cylinder containing the lamina was separated from the myelin transition zone and saved. A second cylinder, 1.5mm in length, directly behind the lamina and through the myelin transition zone, was cut and discarded. The preceding 4.0mm long nerve sample was saved and labeled as myelinated optic nerve (MON). Tissue acquired; lamina- three biological replicates, each containing three samples, and myelinated optic nerve (MON)- three biological replicates, each containing three samples. Tissues were placed in Eppendorf tubes that were flash frozen in liquid nitrogen for 10 minutes prior to storing at -80°C.

The extraction of proteins was conducted by homogenization of tissue in ice-cold tissue protein extraction lysis buffer (T-PER, Pierce Biotechnology) supplemented with protease inhibitor (Sigma- Aldrich), 1 mM phenylmethylsulfonyl fluoride (PMSF), 1 mM Na3VO4, and 1 mM NaF. The lysate was aggitated for 1 h at 4°C followed by centrifugation at 12,000 × g for 10 min at 4°C to clear the cellular debris. The protein concentration was measured using a Bradford assay kit (Bio-Rad). Protein samples were boiled for 10 mins with 1x Laemmli sample buffer and 0.1% β-mercaptoethanol, resolved on 12% sodium dodecyl sulfate (SDS)-polyacrylamide gel electrophoresis (PAGE) and transferred to nitrocellulose membranes. The transferred membrane was blocked for 1 h at room temperature with TBS buffer containing 5% non-fat dry milk followed by incubation with rabbit anti-AQP4 primary antibody (1:1000, Alamone labs) overnight on a shaker at 4°C. The bound primary antibody was detected with a peroxidase-conjugated anti-rabbit secondary antibody (HRP Linked Whole Ab, Sigma Aldrich, Cat # NA934VS, 1:5000). The immunoblot membrane was subsequently stripped and re-probed with and mouse anti-β-actin primary antibody (1:5000, Santa Cruz, to verify equal protein loading), which was detected with a peroxidase-conjugated anti-mouse secondary antibody (HRP Linked Whole Ab, Sigma Aldrich, Cat # NA931VS, 1:5000). Immunoreactive proteins were developed with SuperSignal West Pico plus Chemiluminescence Substrate kit (Pierce, Rockford, IL) and then exposed to X-ray film according to manufacturer’s instructions.

### Statistical analysis

Data were tabulated and compared between treatment groups as mean ± standard deviation or median values. Statistical testing was performed with paired or unpaired t tests for normally distributed data or Wilcoxon rank sum tests for data failing normality testing (GraphPad Prism Version 8, GraphPad Software Inc., La Jolla, California, USA), with significance level of p ≤ 0.05.

## Results

### Immunolabeling

**Fig 1:**
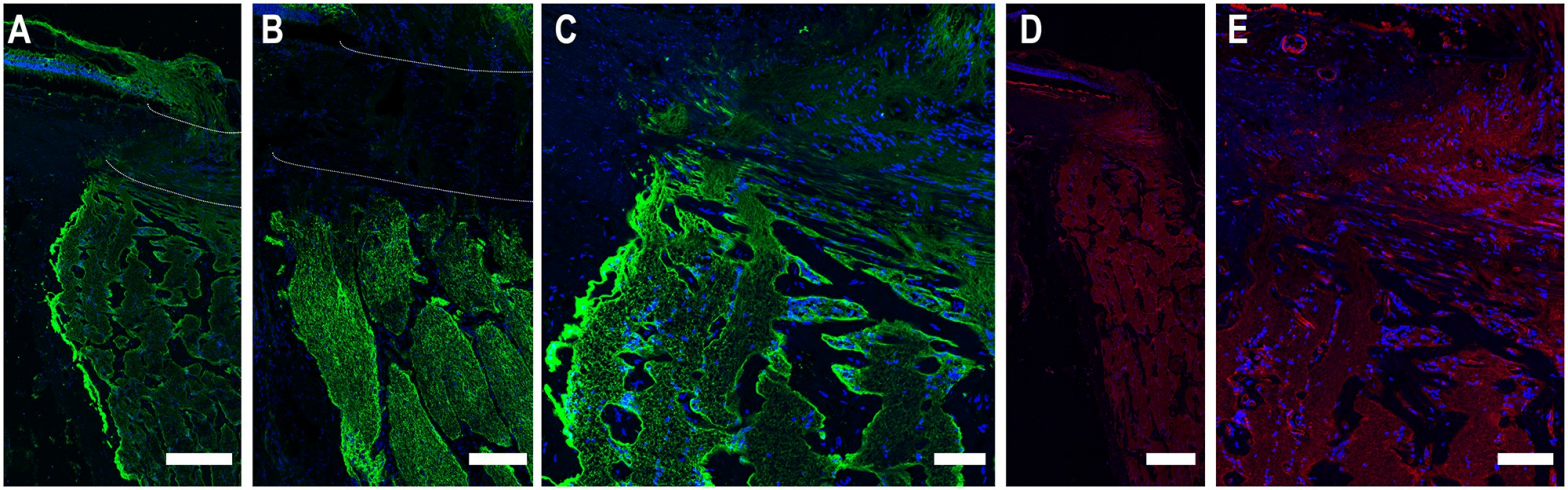
Longitudinal cryosections of immunolabeled aquaporin-4 and phalloidin on human (A-E), and porcine (B) optic nerve head tissue. Minimal label for AQP4 (green) is visible in the lamina cribrosa (between dotted white lines in A, B; seen at higher power in C and E. AQP4 label is prominent in the retinal nerve fiber layer, prelaminar area and myelinated optic nerve in each species. Phalloidin labeling of F-actin (red) is prominent in the prelamina, lamina and myelinated ON (D, E). AQP4 labeling is visible within axon bundles and pronounced along the edge of axons bundles and at the pia surface. DAPI (blue) identifies cell nuclei. Scale Bar: 200 μm (A, D), 50 μm (B, C, E).

**Fig 2:**
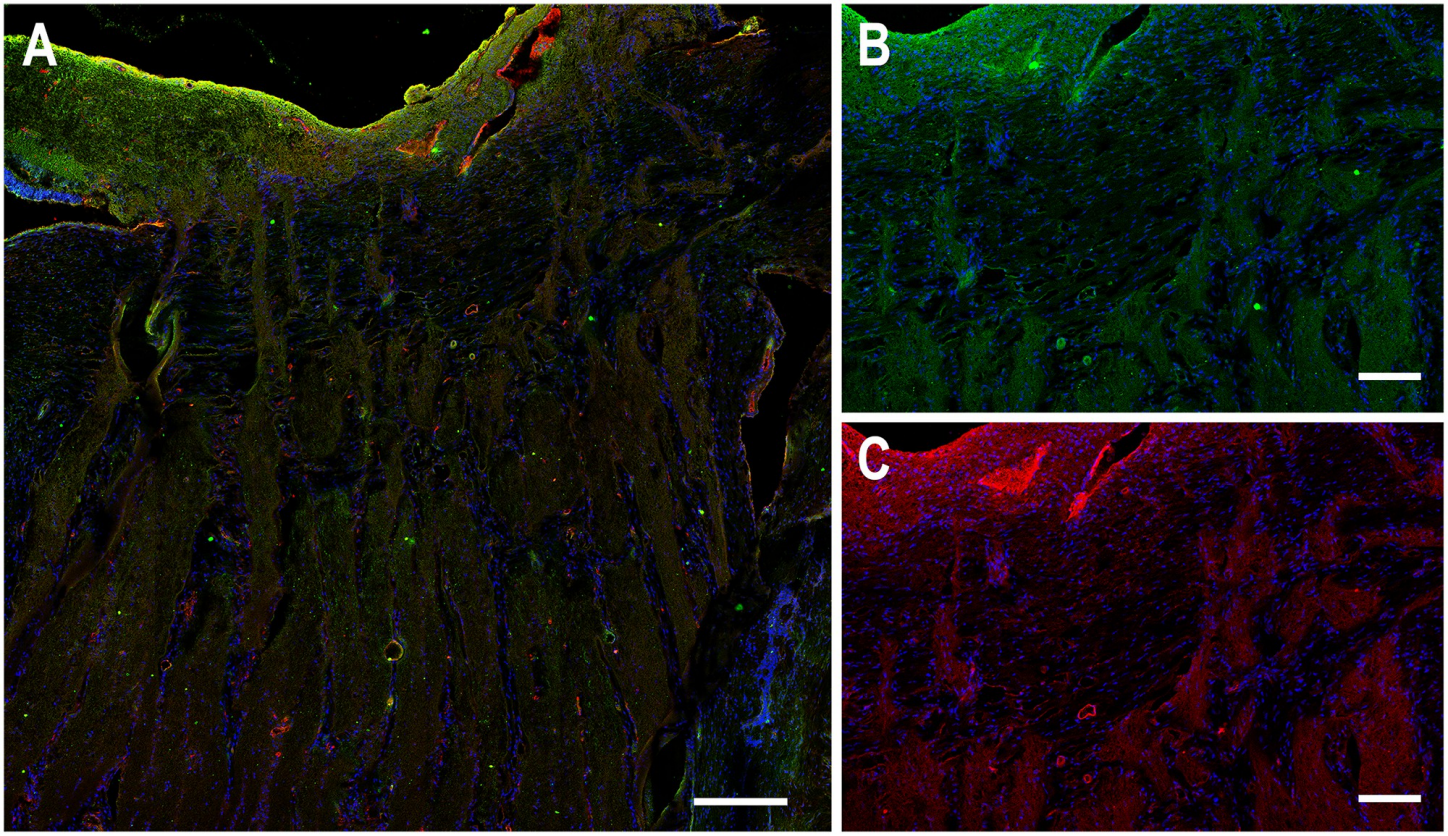
Longitudinal sections of porcine optic nerve head labeled for aquaporin 1 (AQP1, red) and aquaporin-9 (AQP9, green) and DAPI (blue). Labeling of AQP1 and AQP9 was substantial at the internal limiting membrane (A), but minimal throughout the lamina and myelinated optic nerve, except in the walls of larger blood vessel walls as seen in higher power images (B, C). Scale Bar: 100 μm (A), 50 μm (B, C).

Confocal images of longitudinal and cross-sections of human and porcine ONH cryosections were studied with immunolabeling by various antibodies. First, AQP4 labeling was found in the retinal nerve fiber layer, prelamina, and in the myelinated optic nerve (MON), but minimally at the lamina cribrosa region of both species (Fig 1). Labeling was bright at the basement membrane and more prominent at the periphery of nerve bundles in both species than in the more central areas (Fig 1). In the retina, labeling of AQP1 and AQP9 was seen at the internal limiting membrane (Fig 2), but there was no labeling for either AQP1 or AQP9 in neuronal tissue of the lamina cribrosa or the myelinated optic nerve. AQP1, AQP4, and AQP9 labeling was also present in the immediate region of blood vessels.

Phalloidin labeled the F-actin in astrocytes and axons in the optic nerve head, as well as fibroblasts in the peripapillary sclera in both porcine and human tissue. At the lamina cribrosa, the pattern of actin labeling was both linearly from side to side across the nerve head (astrocytes) and parallel to the long axis of the optic nerve within axonal bundles. (Fig 1D and 1E). Phalloidin also labeled the walls of blood vessels and capillaries.

**Fig 3:**
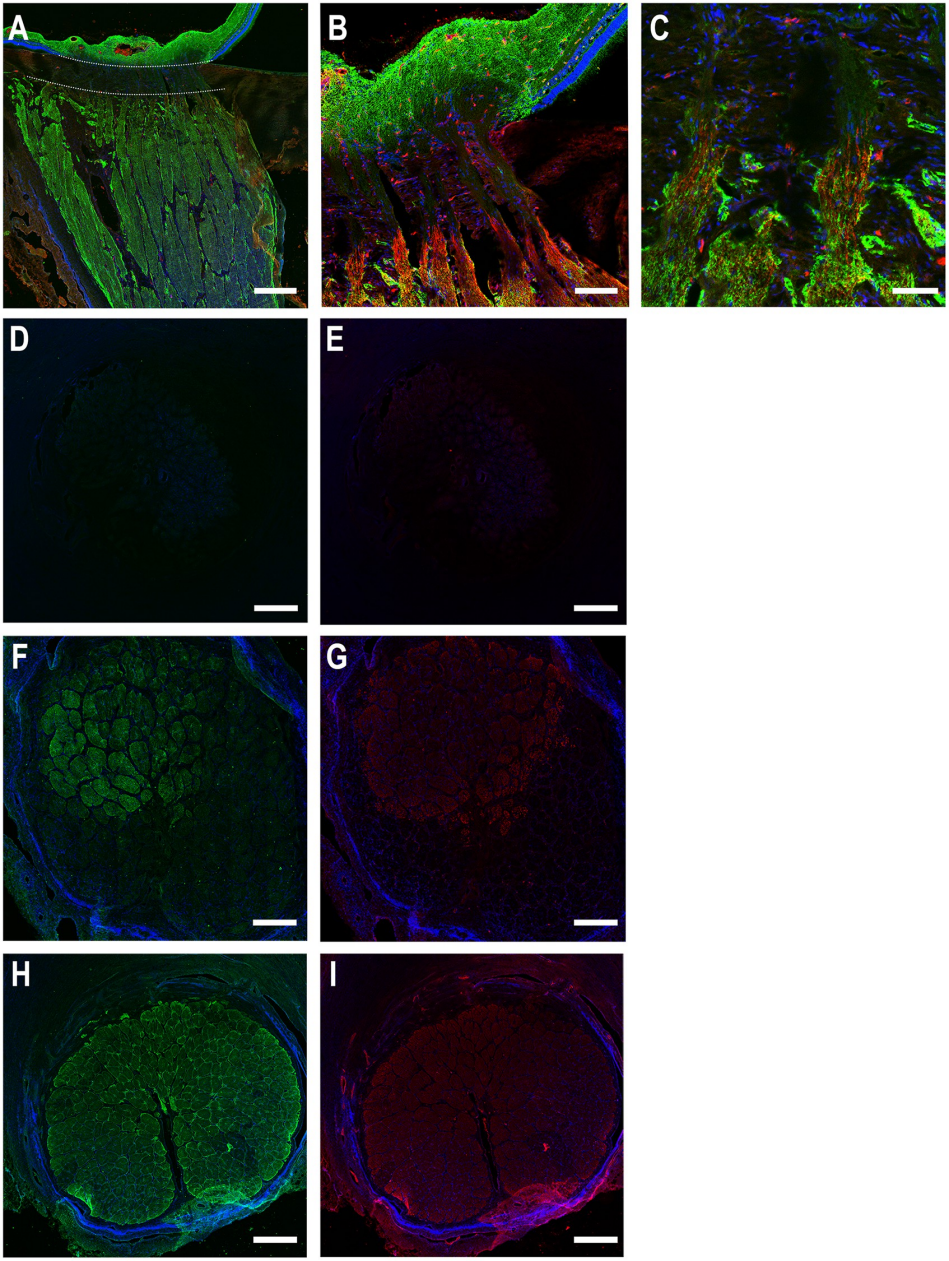
Longitudinal (A-C) and cross-sections (D-I) of porcine optic nerve head labeled for aquaporin 4 (AQP4, green), myelin basic protein (MBP, red) and DAPI (blue). In longitudinal section, A, the lamina cribrosa is indicated by the zone between dotted white lines. Label for AQP4 is present in the retina and prelaminar region, absent in the lamina, and begins again coincident with the initial zone of MBP labeling. The lamina cribrosa in cross-section (D,E) is devoid of both AQP4 and MBP. In F and G, the section has lamina in the inferior area and the initial myelinated optic nerve present in the upper portion, showing that MBP staining begins just anterior to that of AQP4. The myelinated optic nerve (H, I) labels for both AQP4 and MBP. Scale Bar: 200 μm (A, D, E, F, G, H, I), 50 μm (B), 25 μm (C).

Myelin basic protein (MBP) labeling was present in the axon sheaths in the myelinated optic nerve. Its initial appearance in longitudinal sections began just anterior (toward the retina) from initial AQP4 staining in the myelinated optic nerve (Fig 3A–3C). In cross-sections of the porcine optic nerve head (Fig 3D–3F), it can be seen that there was no labeling of either AQP4 or MBP in the lamina cribrosa. As sections sequentially included areas of MBP positivity, AQP4 positivity also appeared. MBP positivity was also seen in some blood vessels through the ONH.

**Fig 4:**
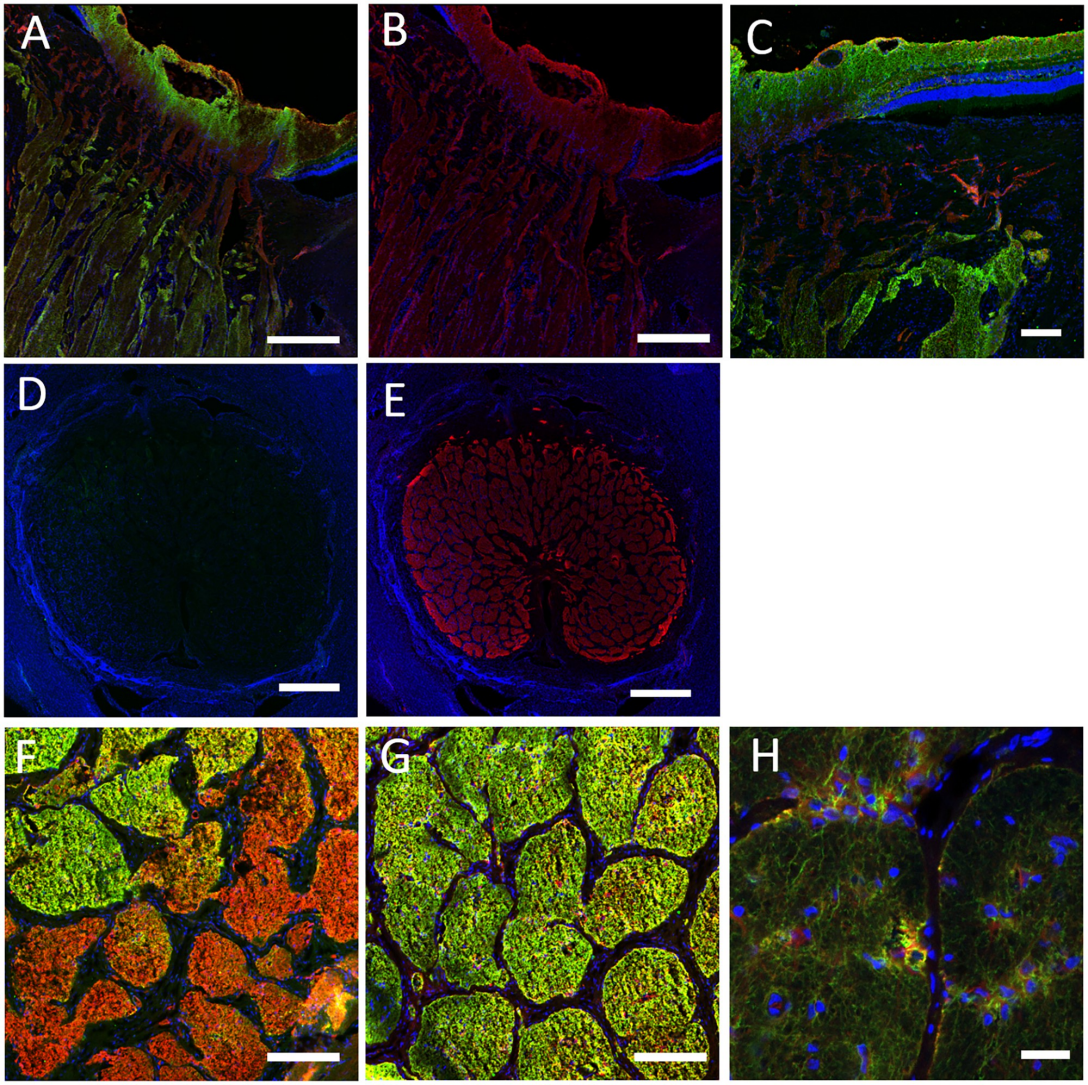
Longitudinal (A, B) and cross-sections (C-F) of porcine tissue labeled for aquaporin 4 (AQP4, green), glial fibrillary acidic protein (GFAP, red) and DAPI (blue). Labeling for GFAP is visible throughout (A-C), in retina through the lamina and into the myelinated nerve, while AQP4 is only visible in the retina and myelinated nerve. Cross-sections of the lamina region (D, E) show label for GFAP but not for AQP4. Higher power cross-section of the transition zone from lamina to myelin shows co-incident labeling for GFAP and AQP4 in central lamina (F). In myelinated nerve, both labels are present, but are somewhat more distinct in position from each other (G). High power image (40x) shows the GFAP and AQP4 co-localization (yellow/orange) in astrocytic cells within and along the periphery of nerve bundles. Scale Bar: 150 μm (A, B), 50 μm (C, D, E), 25 μm (F, G) 10 μm (H).

Astrocytes of the optic nerve head constitutively expressed the intermediate filament protein, glial fibrillary acidic protein (GFAP, Fig 4). GFAP label was also present in retinal Müller cells (Fig 4B) cells. Astrocytes labeled for GFAP in the prelamina, lamina, and myelinated optic nerve (Fig 4). It was noteworthy that GFAP labeling, but not AQP4 label was present in the lamina cribrosa. GFAP label was particularly evident at the junction of lamina with both the sclera and the pia mater.

**Fig 5:**
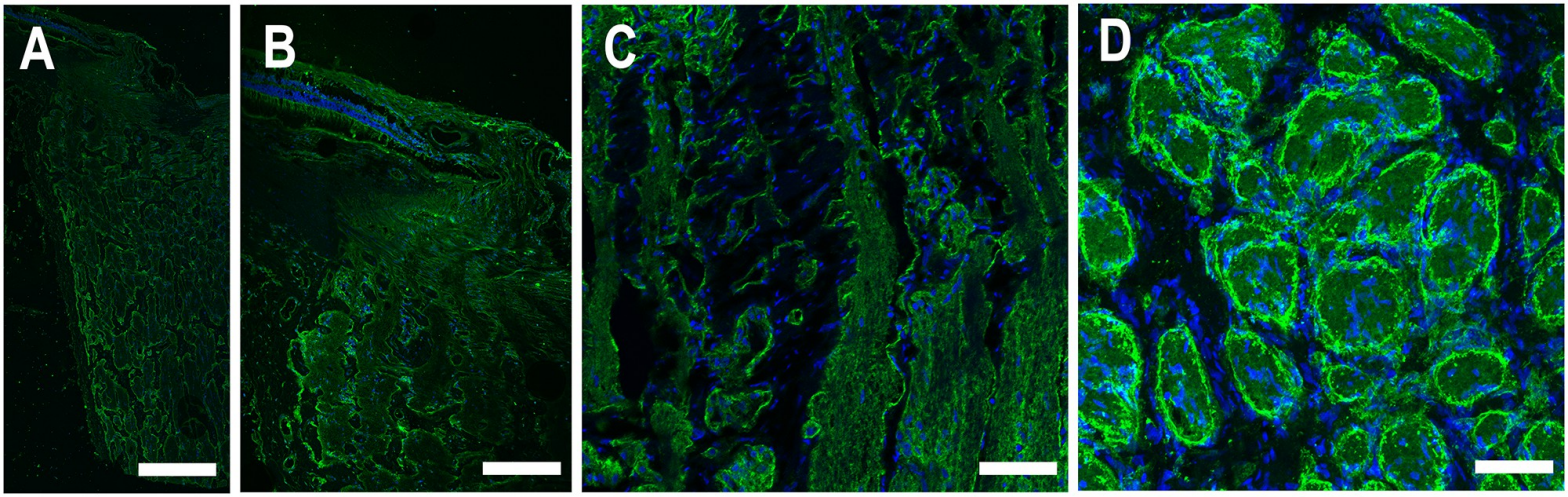
Labeling for α-dystroglycan (αDG, green) in human (A, B) and porcine (C, D) optic nerve head region in longitudinal sections (A, B, C) and cross-section (D). αDG is present throughout the retina, pre-lamina, lamina, and myelinated nerve. It is more evident in the periphery of axon bundles, basement membrane and bordering blood vessels. DAPI (blue). Scale Bar: 200 μm (A), 50 μm (B), 25 μm (C, D).

Alpha-dystroglycan (αDG) is a membrane-bound protein whose presence is essential for the formation of AQP channels. We next evaluated whether the absence of AQP4 in the lamina was associated with a local lack of αDG. αDG was present throughout the glial cells of the retina, lamina and myelinated nerve (Fig 5). Specifically, it was clearly present in astrocytes of the lamina cribrosa (Fig 5B). αDG labeling was more prominent at the periphery of axon bundles and adjacent to blood vessels in both longitudinal and cross-sections (Fig 5C and 5D). αDG also lined the lumen of blood vessels.

### Semi—Quantification of regional aquaporin 4 labeling

**Fig 6:**
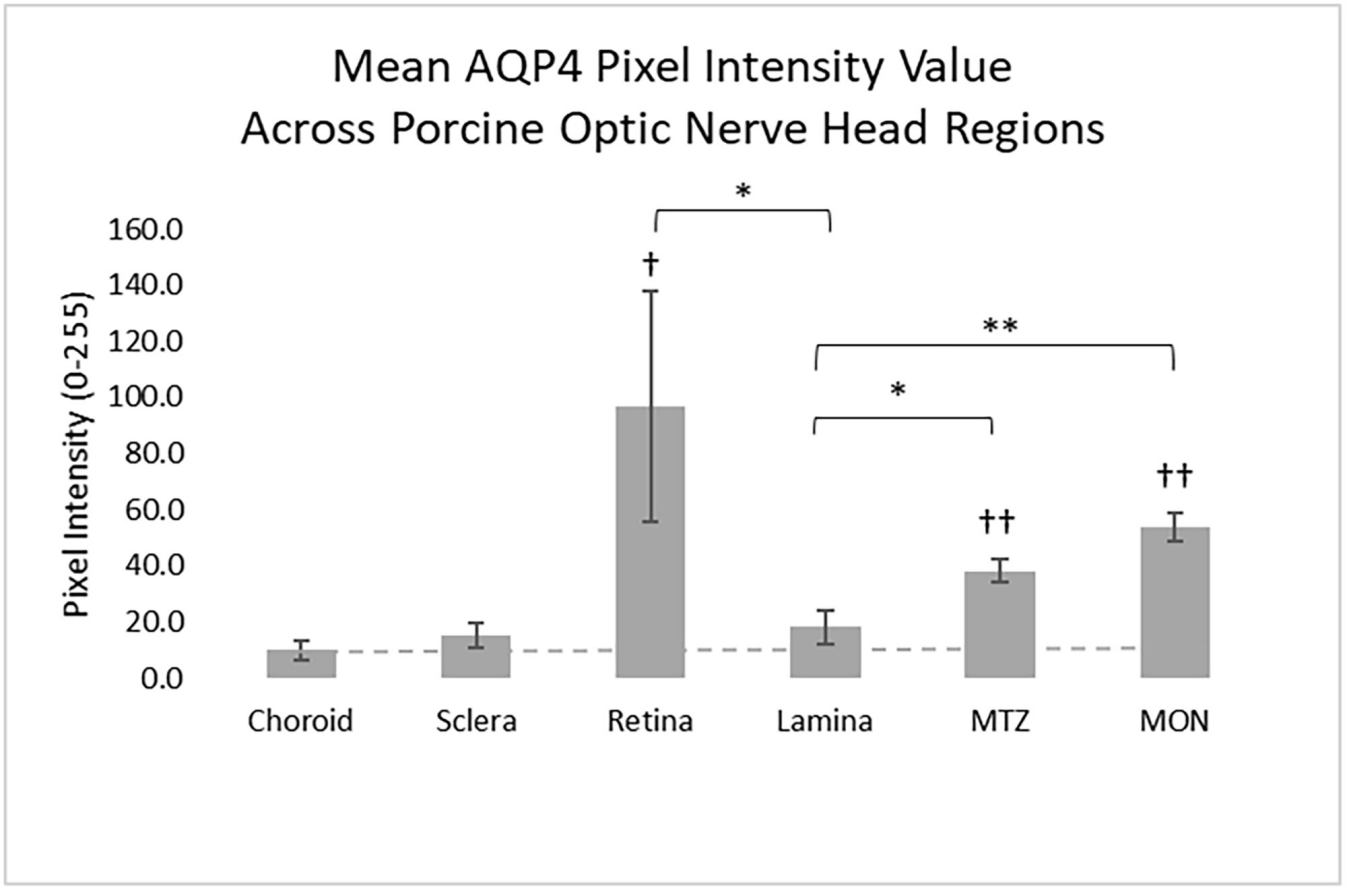
Semi-quantification of aquaporin-4 labeling in 6 regions of porcine ONH region. Values in choroid, sclera, and lamina are minimal, not significantly different from each other, while each was significantly lower than retina, myelin transition zone (MTZ), and myelinated optic nerve (MON). Mean ± standard errors. Dotted line identifies the AQP4 background level in choroid. †p < 0.05, ††p < 0.01 for difference from control choroid. *p < 0.05, **p < 0.01 for difference from lamina.

We quantified the labeling of AQP4 in porcine nerve head zones in units of pixel intensity values (PIV) from 6 areas of the optic nerve head region. The highest AQP4 pixel intensity was in the retina and the lowest was in the lamina cribrosa, sclera and choroid. AQP4 PIV in retina was 96.6 ± 69.6, significantly higher than lamina cribrosa (18.2 ± 11.9), choroid (9.9 ± 6.2) and sclera (15.0 ± 10.0; all p < 0.05, t-tests, Fig 6). The AQP4 level in the lamina cribrosa was not statistically different from sclera (p = 0.70) or choroid (p = 0.27, t-tests), which lack astrocytes and are therefore a tissue background control. AQP4 labeling was significantly higher at the myelin transition zone just behind the lamina, and higher in the myelinated nerve than in the lamina (38.2 ± 10.6, 53.9 ± 13.8, respectively; p<0.05 and p<0.01, Fig 6). Immunogold staining and imaging via transmission electron microscopy have shown AQP4 expression in mouse brain endothelial cells [40] and rat retina endothelial cells [18]. Both found the expression at levels lower than astrocytes. Since the lamina cribrosa in pig and human eyes have small and large blood vessels, the minimal labeling for AQP4 and in our qPCR data is likely to be due to this component (see S1 Fig).

### Regional gene expression

**Fig 7:**
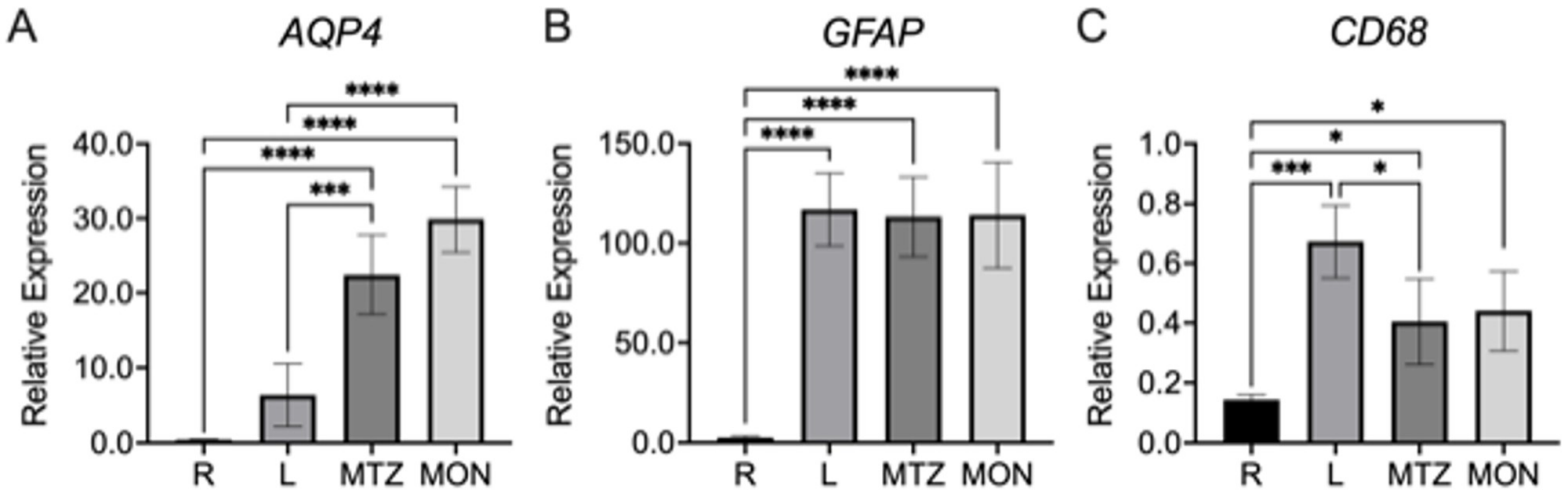
Relative mRNA expression of *AQP4* (A), *GFAP* (B), and *CD68* (C) in regions of the porcine retina and optic nerve head. All samples were normalized to the geometric mean of the corresponding housekeeping gene values. R, Retina; L, Lamina; MTZ, myelin transition zone; MON, myelinated optic nerve. N = 4 replicates per region. Standard deviation error bars. Data was considered statistically significant if p < 0.05; * (0.033), ** (0.0021), *** (0.0002), **** (0.0001).

We next measured gene expression levels of *AQP4* in porcine retina and ONH regions by qPCR. Consistent with the quantitative immunolabeling data, the lamina had significantly lower *AQP4* expression compared to both myelin transition zone and myelinated nerve (Fig 7A). The highest *AQP4* mRNA levels were detected in the MON, but there was no statistically significant difference in expression between MTZ and MON regions (Fig 7A). While the retina had the highest AQP4 pixel intensity by immunolabeling, *AQP4* mRNA expression in whole retina was lower than the other tissue regions (Fig 7A). This likely resulted from the fact that the qPCR retinal specimens contained whole retina, in which the glial components would be a small minority of all cells, while the AQP4 immunolabeling data came from the immediate prelaminar retina, consisting mostly of axons and astrocytes. This interpretation is also consistent with the significantly lower *GFAP* mRNA that we observed in retina than the optic nerve (Fig 7B). There were no significant differences in *GFAP* mRNA expression across the lamina, myelin transition zone, and myelinated nerve, which was consistent with GFAP immunolabeling (Fig 7B). Conversely, *CD68*, a macrophage/microglial marker, was highest in the porcine lamina (Fig 7C). The retina tissue had detectable, but substantially lower expression of *CD68* (Fig 7C).

### Western blot

**Fig 8:**
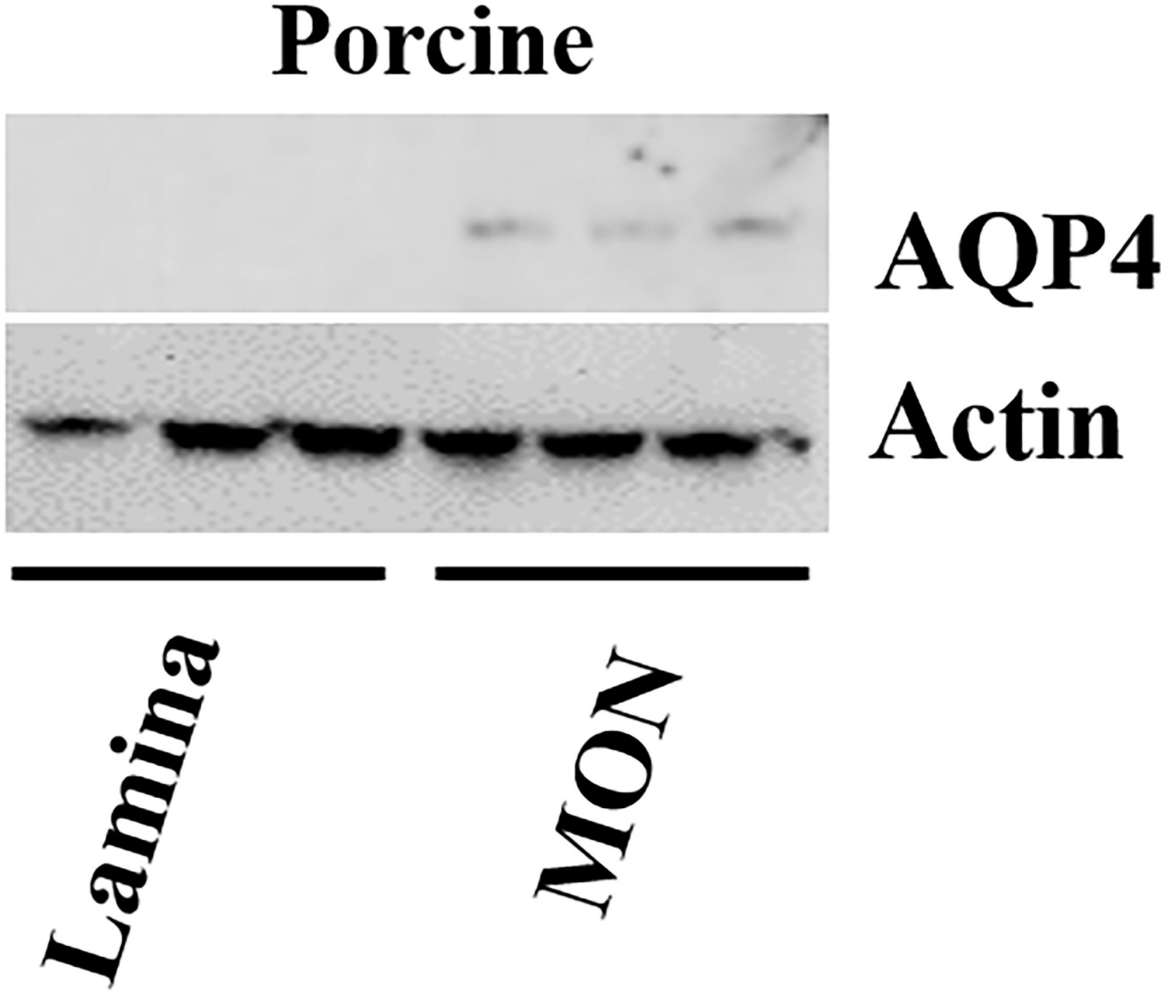
Western blot of aquaporin-4 (AQP4) on lamina and myelinate nerve (MON) of porcine tissue. Three biological replicates with three samples of lamina (lane 1,2,3) and three biological replicates with three samples of MON (lane 4,5,6) were processed. AQP4 was only detected in the MON samples, while none detected in the lamina tissues. All 6 lanes were positive for β-actin.

Protein presence of AQP4 in porcine tissue was studied via western blot analysis. AQP4 protein expression was present in three MON replicates, but no detectable AQP4 protein was found in any of the three lamina samples (Fig 8). Actin was used a positive control for all six samples, and indeed showed positive labeling and equal protein loading.

## Discussion

Our results reported here showing the lack of significant expression of AQP4 in the lamina cribrosa of human and porcine eyes is consistent with our previous findings with mise and rats and suggests the more universal finding, that these channels are likely not present in the equivalent of the lamina cribrosa in any mammalian eye. The minimal presence of AQP4 at the large animal lamina and the unmyelinated nerve of rodent eyes is highly evolutionarily conserved and therefore may be either advantageous for normal ONH function, and/or a protective influence for retinal ganglion cell axons from glaucoma damage [39]. There are several possible hypotheses for the potential benefit from this regional astrocytic characteristic.

First, the lack of AQP4 at ONH could reduce local tissue swelling or contraction by preventing imbibition or loss of water in astrocytes. Our recent quantitative studies in mouse ONH show that the astrocytic component occupies nearly three-fourths of ONH volume [31]. While the large mammal ONH has additional connective tissue beams, the astrocyte fraction still occupies nearly half of the ONH volume normally (work in progress). The inability to expand in the closed ONH compartment, could prevent astrocytes could from enhancing the axonal transport obstruction in axons known to occur in glaucoma. We showed that the myelinated optic nerve in normal mice is larger in area than in AQP4 null mice, due to a reduced astrocytic volume in the nulls [39]. Previous research showed that increased IOP leads to greater fluid movement from the vitreous cavity through the ONH [41]. Thus, the absence of AQP4 channels would avoid detrimental intracellular water intake and tissue swelling in the presence of increased IOP.

A second potential beneficial factor for the lack of AQP4 at the mouse unmyelinated nerve and the lamina cribrosa of larger mammals relates to the fact that astrocytes of this zone are uniquely subjected to asymmetrical mechanical forces from hoop stress generated in the peripapillary sclera and the translaminar pressure gradient between IOP and optic nerve tissue pressure. To our knowledge, few other astrocyte types in the central nervous system has a similar need to mechanically sense stress and to respond to it in a manner that preserves normal neuronal structure and function. Hypothetically, the lack of water ingress from the minimal presence of AQP channels could permit a more robust cytoskeletal support of the ONH that might be compromised by intracellular volume expansion.

Finally, it is known that the blood—brain barrier is defective in the ONH at the level of the choroid [42]. At this location, cytokines and other proteins have some access to the ONH. Without AQP4 channels to imbibe fluid, extracellular diffusion out of the lamina may be accelerated, facilitating the removal of such chemical and protein elements from the ONH. If this mechanism is operative, it would potentially be protective of retinal ganglion cell axons. The exact rationale for minimal AQP4 in the ONH is unknown, nonetheless, it is a particular phenotypic feature of the astrocytic cells in this region.

In addition to the relative absence of AQP4 at the ONH, we have identified other regionally unique features of the ONH astrocytes in porcine and human, including the presence of electron dense, junctional complexes at the interface between astrocytes and their basement membranes [32]. In the brain, retina, and myelinated optic nerve, astrocytes contact capillaries and other vessels or the brain surface at all of their processes, but do not form a basement membrane to contact connective tissues. By contrast, mouse ONH astrocytes and those in the porcine and human lamina, are widely in contact with the dense connective tissue of the peripapillary sclera and of lamina cribrosa beams. As a result, their structure differs from astrocytes in various regions that reside within the dense network of unmyelinated axons, dendrites and glia cell processes. We speculate that the components of these junctional complexes are instrumental in their mechanical interaction between the stresses of IOP transmitted from the sclera and via the translaminar pressure gradient. In recent experimental mouse glaucoma, we detected that loss and disconnection of the junctional complexes was coincident with separation of astrocytes from their basement membranes [32]. Further study is merited to denote the components of these complexes and their potentially translational role in glaucoma treatments.

Astrocytes are the most numerous glia cell in mammalian ONH [43] and the reaction of astrocytes to injurious stimuli can be either beneficial or detrimental [44–46]. Features categorized as beneficial include activation of the JAK-STAT pathway [47], and STAT knockout in mouse glaucoma leads to greater ganglion cell loss [6]. The detrimental astrocyte behavior may arise through stimulation from microglia with interleukin 1α, tumor necrosis factor α (TNFα), and complement component subunit 1q (C1q). Inhibition or knockout of TNFα [48] or C1q [49] reduce experimental glaucoma loss of ganglion cells. This suggests that both types of astrocyte behavior may be involved in the response to glaucoma and that the relative equilibrium between them may help to determine the degree of damage. Nutritional support to axons from astrocytes is another potential contributing feature of their behavior that can impact neurodegeneration [50, 51].

The existence of a glymphatic pathway for fluid exit from the brain through astrocytes was proposed by Iliff et al. [52], based on movements of various solutes. While not disagreeing that there is extracellular solute movement, Verkman et al. challenged the interpretation of these data and the existence of an astrocyte-dependent pathway [53]. This controversy continues in studies of brain [54, 55]. Tracer studies by Matthieu et al. [30] showed appearance of dyes in the mouse myelinated optic nerve after cisternum magnum injection, but did not find astrocytic cells to be explicitly responsible for the movement, which would be unlikely. since AQP4 channels only transfer water molecules, not molecules of the size of these experimental tracers. While tracer molecular movement is altered in the myelinated optic nerve in mouse glaucoma eyes with significant axonal degeneration, the cellular and extracellular content of atrophic optic nerves is substantially different from normal, leaving several explanations for changes in solute movement.

It has been proposed that abnormal glymphatic pathway behavior could be a contributing feature of glaucoma damage [56]. If such a pathway exists, and if it depends upon AQP4 channels as proposed, our findings in porcine and human eyes, combined with previously published data in mice, do not support this hypothesis. All mammalian eyes so far studied have an absence of AQP channels in the ONH, confirmed here for the first time in porcine and human eyes. The regionally unique nature of astrocytes of the ONH, both structurally and functionally, differs from astrocytes residing both 100 micrometers away from them in both the prelaminar and myelinated optic nerve. The specific phenotype of these glia is integral to both normal ONH function and to the pathological events at the center of glaucoma damage. It is now increasingly recognized that astrocytes have regional specificities that are important to their local functions in the central nervous system [57, 58].

## Supporting information

S1 Fig A longitudinal cryosection immunolabeled for aquaporin-4 (AQP4, green) on porcine (A,B) optic nerve head tissue show actin positive labeling (Phalloidin, red). Phalloidin staining highlights actin in cells’ cytoskeleton, and in addition, it labels blood vessels including this large artery in the pre-lamina and lamina region. AQP4 labeling is visible on the endothelia cells adjacent to lumen of the vessels (white arrow). DAPI (blue) identifies cell nuclei. Scale Bar: 200 μm (A), 50 μm (B).
(TIF)
