# Supplementary figures and images for: Aquaporin 4 is not present in normal porcine and human lamina cribrosa
Source: PLoS One. 2022 Jun 16;17(6):e0268541. doi: 10.1371/journal.pone.0268541 (PMC9202842; doi:10.1371/journal.pone.0268541)

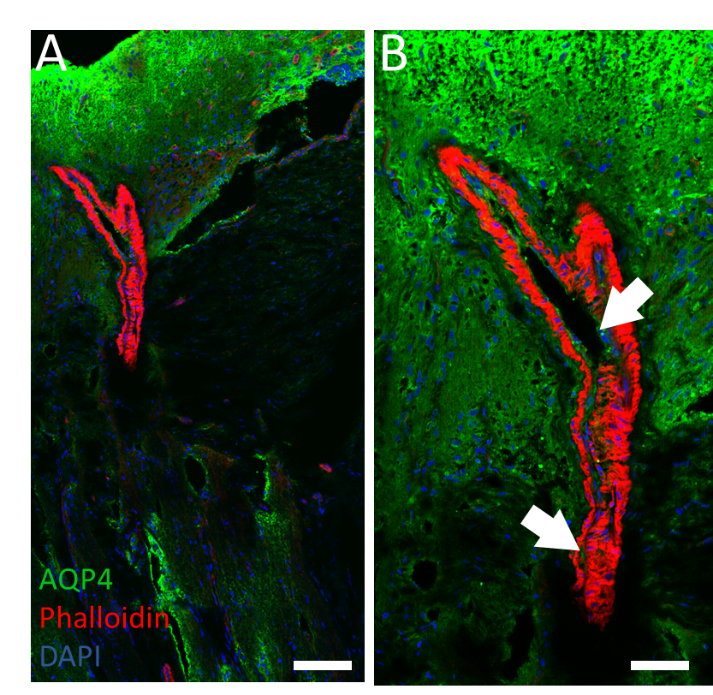

Supplement: S1 Fig — Phalloidin staining highlights actin in cells’ cytoskeleton, and in addition, it labels blood vessels including this large artery in the pre-lamina and lamina region. AQP4 labeling is visible on the endothelia cells adjacent to lumen of the vessels (white arrow). DAPI (blue) identifies cell nuclei. Scale Bar: 200 μm (A), 50 μm (B). (TIF) [file pone.0268541.s001.tif]
